# Striate Artery Infarct After Bilateral Carotid Artery Ligation (BCAL) in a Dog: A Multimodal MRI Study

**DOI:** 10.3389/fvets.2020.580256

**Published:** 2020-09-18

**Authors:** Lukas Komornik, Ines Lautenschläger, Alessio Vigani, Claudia Iannucci, Antonio Pozzi, Adriano Wang-Leandro, Katrin Beckmann

**Affiliations:** ^1^Department of Small Animal Surgery, Small Animal Clinic, Vetsuisse Faculty Zurich, University of Zurich, Zurich, Switzerland; ^2^Department of Diagnostics and Clinical Services, Clinic for Diagnostic Imaging, Vetsuisse Faculty Zurich, University of Zurich, Zurich, Switzerland; ^3^Department of Small Animal Emergency and Critical Care, Small Animal Clinic, Vetsuisse Faculty Zurich, University of Zurich, Zurich, Switzerland; ^4^Department of Neurology, Small Animal Clinic, Vetsuisse Faculty Zurich, University of Zurich, Zurich, Switzerland

**Keywords:** BCAL, stroke, arterial spin labeling, perfusion imaging, angiography

## Abstract

Bilateral carotid artery ligation has been reported as a lifesaving procedure to control severe hemorrhage. However, reports are sparse and little information is available regarding the potential risks associated with this procedure. We report an ischemic brain infarct as a complication after vascular surgery. A 3-year old, male intact border collie was presented for acute onset of forebrain signs 5 days after bilateral carotid artery ligation. Multimodal brain MRI including morphologic sequences, MR angiography, diffusion- and perfusion-weighted images were performed. MRI revealed a well-defined intra-axial lesion of the left caudate nucleus, with increased T2 and decreased T1 signal intensity and moderate heterogeneous peripheral contrast enhancement. The cerebral blood flow was reduced relative to the contralateral caudate nucleus. Images were consistent with a subacute lacunar ischemic infarct of the left striate artery. Additionally, multiple arterio-arterial anastomosis arising from the vertebral arteries were visible in the angiography sequences. Ischemic infarct due to thromboembolism should be considered as a possible complication associated with bilateral carotid artery ligation. Collateral blood supply can develop as early as 5 days after surgery.

## Introduction

BCAL is a lifesaving surgical procedure indicated in dogs with oral or maxillo-facial bleeding that cannot be stopped with standard hemostasis techniques (1–7). Ligation of both carotid arteries in dogs is possible because of a sufficient arterio-arterial collateral blood supply from the vertebral arteries (5, 8). While experimental studies documented adequate blood supply to the brain and no neurological side effects in healthy dogs undergoing BCAL (1, 3, 6, 9), little is known about outcome of clinical patients after this procedure. Reported complications in dogs after carotid artery surgery include cerebral edema and retinal damage (10). In humans increased risk of perioperative strokes have been observed in patients undergoing carotid artery and heart surgery (11). In dogs, carotid artery and heart surgeries are not as common as in humans and the risk of perioperative strokes has not been investigated.

We report a striate artery infarct in a young adult Border collie 5 days after BCAL due to uncontrollable traumatic maxillofacial bleeding.

## Case Presentation

A 3-year-old, male intact Border Collie was presented to the Veterinary Hospital of the Vetsuisse Faculty Zurich for profuse acute epistaxis as well as oral hemorrhage, occurring after playing with a stick. The origin of the hemorrhage could not be determined during oral examination and an underlying coagulopathy was excluded based on point of care clotting tests. For further workup, helical CT of the head and neck was performed under general anesthesia. The examination was performed with a 16-slice scanner (Philips Brilliance16, Philips AG; Zurich, Switzerland) in 1.5 mm slices with the dog positioned in sternal recumbency and the head away from the gantry. Scanning parameters were set at 280 mA, 120 kV, 1 s rotation time, 0.7 pitch. Non-ionic iodinated contrast agent (700 mg I/kg; Accupaque, GE Healthcare AG, Opfikon, Switzerland) was given intravenously, and post contrast scanning was programmed at 60 s after injection. CT showed accumulation of a large amount of heterogeneous soft-tissue attenuating material with few gas inclusions was present in the pharyngeal region, with focal contrast pooling seen immediately medial to the right pterygoid process (Supplementary Figure 1). Therefore, the CT diagnosis was a penetrating lesion of the right pharynx as the primary cause of the active hemorrhage.

The dog underwent emergency surgical exploration to manage the hemorrhage. The bleeding site was surgically inaccessible and the hemorrhage persisted despite local pressure hemostasis. A BCAL was performed as described by Goodman and Goodman (7). The bleeding ceased and the dog recovered uneventfully from the procedure. The dog was discharged 2 days later and was clinically normal with no evidence of neurological deficits. Five days after surgery, the dog developed sudden onset of ataxia, circling to the left, dragging of the right front and hind limb and behavioral changes. Physical examination was within normal limits. Hematocrit was 38% (Ref.: 42–55%), median blood pressure was 134 mmHg (Ref.: 80–100 mHg) measured with non-invasive oscillometer. Rotational thromboelastometry was performed to assess coagulation and results were within normal limits. The neurologic examination revealed behavior changes, slight pleurothotonus to the left, circling to the left and a decreased postural reaction on the right front-limb. The neuroanatomic location was defined as left cerebrum. Because of the hyperacute onset 5 days after BCAL, a vascular insult was suspected, and MRI of the brain was recommended. The owner initially declined MRI and based on the high suspicion of a thrombotic event causing the neurological presentation the dog was anticoagulated with intravenous Heparin Constant Rate Infusion (800 UI/kg/day) for 16 h and Clopidogrel (1 mg/kg SID p.o) to prevent further thrombus formation. Neurological signs slowly improved and 2 days thereafter, an MRI examination of the brain was performed to investigate the cause of the acute onset of central nervous system deficits.

## MRI-Findings

Imaging of the brain was performed with a 3 Tesla MRI scanner (Philips Ingenia, Philips AG, Switzerland) using a 20-channel head/neck coil. The dog was positioned in dorsal recumbency and the scanning protocol included morphological sequences, diffusion-weighted imaging (DWI), magnetic resonance angiography (MRA), and perfusion weighted imaging (PWI) (Table 1). For post-contrast image acquisition, gadopentetate dimeglumine (0.1 mmol/kg; Omniscan, GE Healthcare AG, Opfikon, Switzerland) contrast medium was administered intravenously. Apparent diffusion coefficient maps were computed from DWI. MRA was acquired using time-of-flight and contrast-enhanced angiography; PWI was acquired using 3D pseudo continuous arterial spin labeling (3D pCASL) imaging sequence as previously reported (12).

**Table 1:** Imaging protocol.

**Sequence**	**Plane**	**Repetition time (ms)**	**Echo time (ms)**	**Slice thickness (mm)**
T2-W	Transverse	4820	100	2.5-2.8
FLAIR	Transverse	11000	125	2.8
3D-T1W GE	3D	11	5.1	0.7
SWI	Transverse	31	0	2
DWI	Transverse	3914	98	2
PWI	Transverse	4295	15	6
MRA TOF	3D	4.8	1.8	1.2
MRA CE	3D	23	3.5	1.2

*T2W, T2-weighted; T1W, T1-weighted; FLAIR, Fluid attenuation inversion recovery; T1W, T1-weighted; GE, gradient echo; SWI, susceptibility-weighted; DWI, diffusion-weighted imaging; PWI, perfusion-weighted imaging; MRA, magnetic resonance angiography; TOF, time-of-flight; CE, contrast-enhanced*.

The conventional sequences revealed a well-defined lesion centered within the left caudate nucleus, extending ventrally, mildly laterally and rostrally and involved the basal nuclei (putamen and pallidum) as well as the internal capsule. This lesion was T2- and FLAIR hyperintense and hypointense on precontrast T1 weighted images relative to spared cortical gray matter.

**Figure 1:**
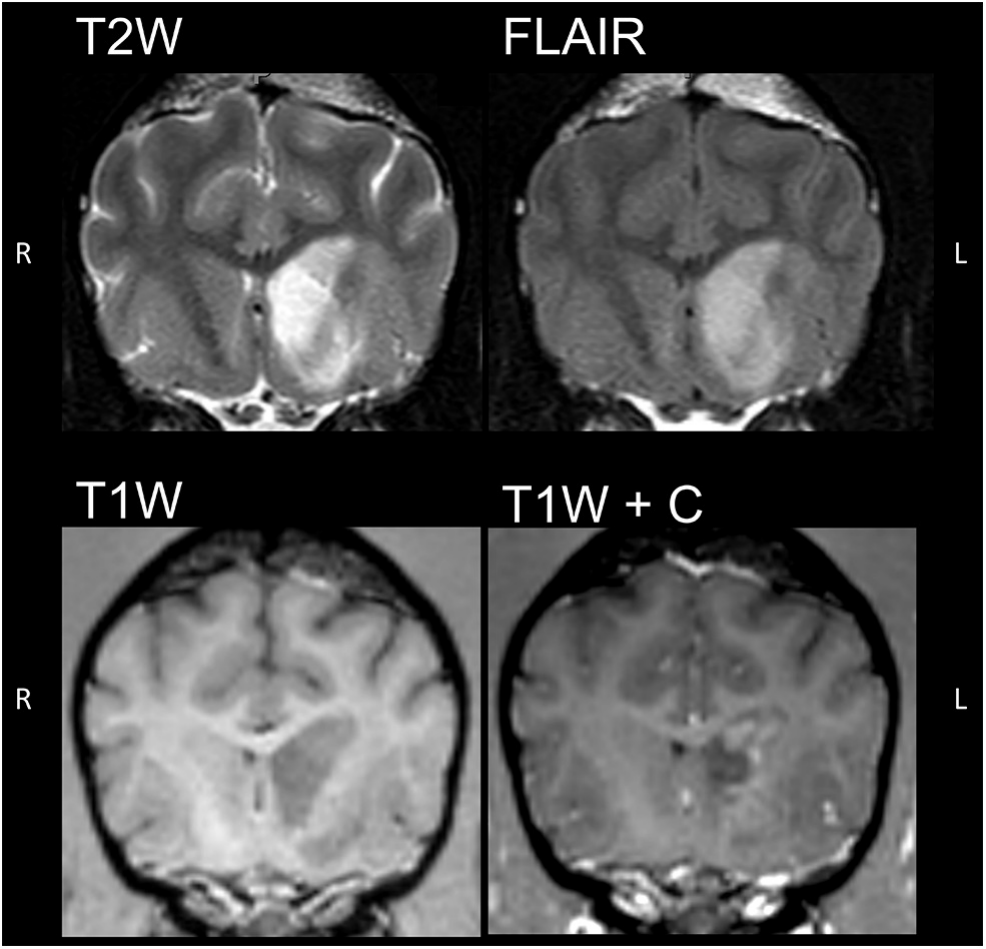
Transverse images of the brain in T2-weighted (T2W), fluid attenuation inversion recovery (FLAIR) and 3D-T1 pre and post contrast sequences at the level of the orbital fissure of the affected dog. A well-defined lesion centered within the left caudate nucleus showing increased T2 and FLAIR and decreased T1 signal intensity, with moderate peripheral contrast enhancement.

Overall, there was no mass effect associated with the lesion except for a mild compression of left lateral ventricle. A thin FLAIR- hyperintense rim outlining the lateral contour of the rostral aspect of the left lateral ventricle was also noticed (Figure 1).

Post contrast T1 weighted images showed moderate, heterogeneous, peripheral, faintly rim-like contrast enhancement and a central non-enhancing core. The sequences sensitive to the susceptibility effects caused by paramagnetic properties of hemoglobin degradation compounds (SWI) showed no signal changes in the described lesion.

**Figure 2:**
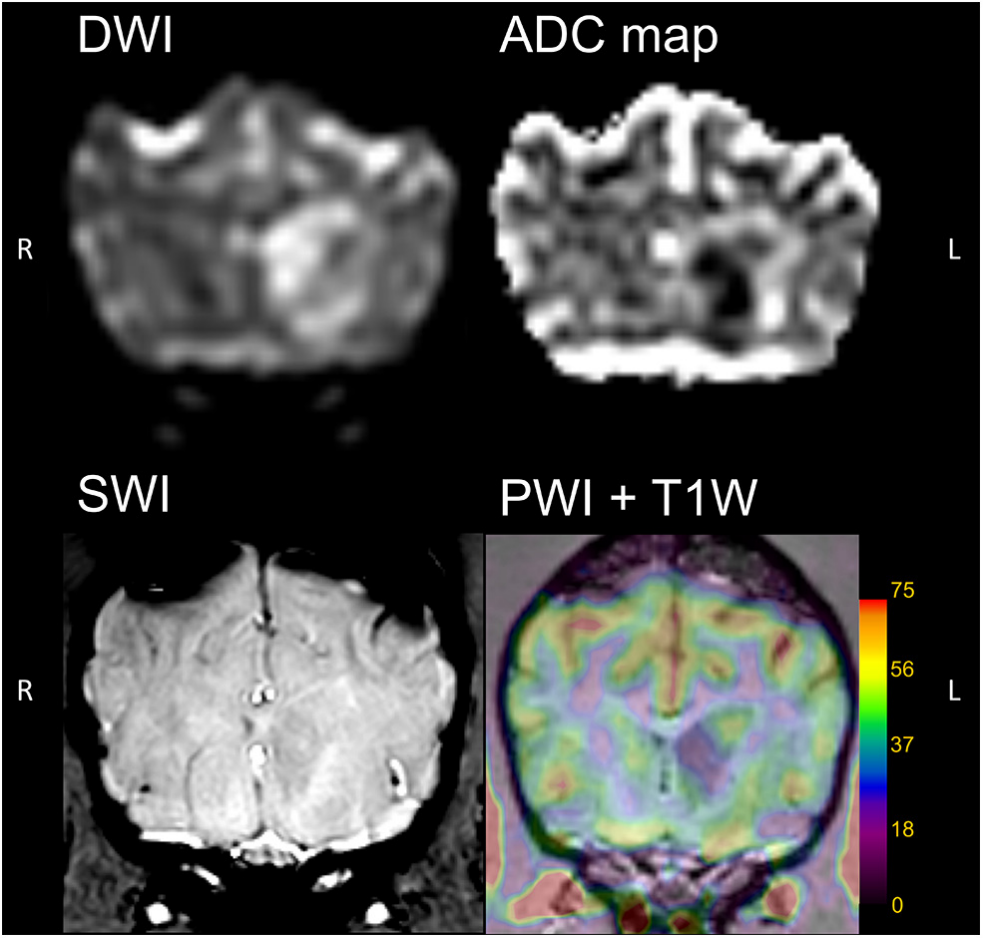
Transverse images of the brain in diffusion-weighted (DWI), computed apparent diffusion coefficient (ADC) maps, susceptibility-weighted (SWI) and perfusion-weighted (PWI) sequences. The lesion in the left caudate nucleus was hyperintense in DWI and hypointense in ADC, consistent with subacute ischemic infarct. Moderate peripheral hyperperfusion is noticed on PWI. No signal void is present intralesional in SWI.

The lesion was hyperintense on the DWI sequence. The corresponding ADC map showed low signal intensity within the lesion core and increased peripheral signal intensity. Perfusion imaging revealed a well-defined, irregularly contoured area of decreased CBF within the left caudate nucleus (Figure 2; CBF left caudate nucleus = 36.3 ml/100 g/min, lesion core = 22.5 ml/100 g/min, right caudate nucleus = 41.4 ml/100 g/min), corresponding to the above mentioned non-enhancing core. Peripheral to the hypoperfused focus, an ill-defined area of increased perfusion was present, matching the patchy peripheral enhancement noticed in the post-contrast anatomical sequence (Figure 2).

**Figure 3:**
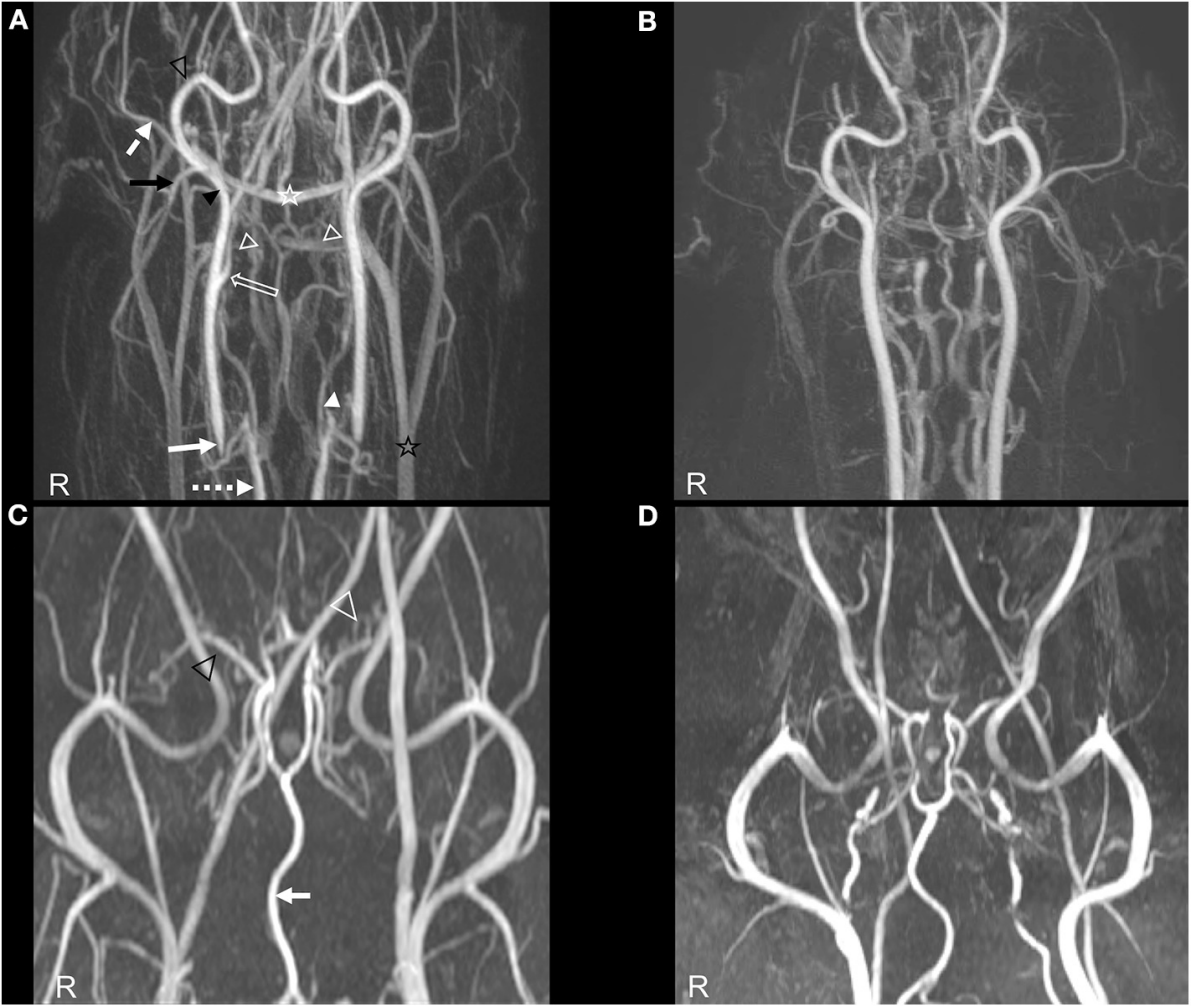
Angiography of the affected dog **(A,C)** compared to a normal dog **(B,D)**. **(A)** TOF- MRA image in dorsal orientation of the patient with indicated relevant arteries and collaterals. White arrow, Tapering right common carotid artery at the level of the ligature; Pointed white arrow, right vertebral artery; Hollow white arrow, right internal carotid artery; Black arrow head, right external carotid artery; Hollow white arrowheads, presumed superficial collateral network between caudal auricular artery and cranial thyroid artery; White arrowhead, presumed deep collateral pathway between both vertebral arteries and the common carotid arteries cranial to their ligation site; Black arrow, caudal auricular artery; Dashed white arrow, superficial temporal artery; Hollow black arrow head, maxillary artery; Hollow white star, Transverse sinus; Hollow black star, External jugular vein. **(B)** TOF- MRA image in dorsal orientation of a normal, size matched dog. **(C,D)** CE- MRA image of the head in dorsal orientation showing the relevant arteries. **(C)** Hollow white arrow head, supernumerary arteries; Hollow black arrow head, right middle cerebral artery. **(D)** CE- MRA image of a normal, breed and age matched dog.

The CE- MRA and TOF sequences of the brain and neck showed the ligation of both common carotid arteries as abrupt tapering of the contrast enhanced arteries at the level of the caudal aspect of the second cervical vertebrae (Figure 3A). The arterial circle of the brain and the branching of both middle cerebral arteries were well-delineated on the CE- MRA sequences. However, the striate arteries could not be identified separately. On both sequences, TOF and CE- MRA, the formation of numerous arterio- arterial anastomoses and collaterals can be identified on MRA (Figures 3A,C). Although the striate arteries could not be clearly identified in MRA, both middle cerebral arteries were well-delineated. Multiple, small, and well-defined branches arising from the left middle cerebral artery could be identified compared to the contralateral and to reported literature of canine brain MRA (13, 14) (Figure 3).

The diagnosis based on MRI was a subacute ischemic infarct of the left striate artery with mild associated perilesional cytotoxic edema and presence of multiple extracranial arterio-arterial anastomoses secondary to BCAL.

## Outcome

Clopidrogrel anticoagulant therapy was converted to acetylsalicylic acid (3 mg/kg SID p.o.) because of financial concerns of the owner and the dog was discharged 1 day after the MRI. At re-check examination 1 week later, right sided postural reaction deficit was still present. Circling to the left was improved, but still intermittently present. Four weeks later the owner reported that the dog was back to normal but less resilient to exercise than before. Ten weeks after first presentation the dog returned to working again as a sheepdog 1,800 meters above sea level without any signs of subsequent damage.

## Discussion

This is the first clinical case report with an MRI diagnosis of an ischemic brain infarct as a complication of BCAL. Studies using dogs as experimental models for BCAL report no neurological deficits, assuming that cerebral perfusion is not affected by the acute vascular ligation (9). The explanation for this finding is the pivotal role of the vertebral artery system in the blood supply to the brain (5, 8) and the more extensive collateral vessels in dogs compared to humans (1, 5). However, redistribution of blood supply through a single main artery predisposes to uneven distribution of flow into the sequential branching of the artery. This phenomenon is referred to as the Coanda effect, in which the jet arterial flow is corresponding to a vascular bifurcation or branching tends to be directed and forced toward one side. This effect occurs even in the presence of a symmetrical bifurcation of a blood vessel. The uneven distribution of arterial flow inevitably induces stagnation of flow in the vascular branch with low flow and hyperperfusion in the other. In the former, there is increased risk of platelet aggregation and thrombus formation with consequent tissue ischemia, in the latter instead the higher jet flow predisposes to collateral vascular bed formation. The Coanda effect has been previously proposed and demonstrated in the pathogenesis of arterial thrombus formation as well as in other neurovascular anomalies as arterial serpentine aneurysms in humans and may also play a role in the presented dog (15, 16).

In humans, decreased CBF has been measured after temporary unilateral balloon occlusion of the carotid artery even in the absence of neurological deficits; nevertheless, this population has an increased risk for delayed cerebral infarction because of a poor flow reserve (17). In dogs, cerebral perfusion has not been measured immediately after BCAL and therefore it is unknown if reduced reserve capacity might increase the risk for developing cerebral ischemia/infarction, when challenged with hypotension, hypovolemia or hypoxia in the perioperative period. In this clinical case BCAL was performed as lifesaving procedure in an unstable patient. Therefore, all the factors outlined above could have caused a large-to-small artery micro embolism and associated reduced CBF could have further diminished the clearance of thromboembolism that have entered the vascular bed (18, 19), increasing the risk of an ischemic infarct. Large to small vessel infarcts affecting the middle cerebral artery and its branches are a well-known entity in experimental dog models of stroke, where emboli are induced at the level of the internal carotid artery (20, 21).

Recently, use of state-of-art MRI sequences for diagnostic workup of vascular diseases in clinical veterinary medicine settings have been reviewed (22). We documented a brain infarct with multimodal MRI which allowed us to show precisely the temporal characterization of the infarct and presence of arterio-arterial anastomosis as a compensatory mechanism after BCAL. Conventional imaging results together with DWI and ADC were consistent with a subacute ischemic striated artery infarct (20). In this case, cerebral perfusion was measured using ASL. This technique uses the protons of the blood as endogenous tracers by applying radiofrequency pulses at the level of the carotid arteries (23–25). In humans delayed arrival of the arterial blood to the brain and reduced CBF has been detected with carotid artery stenosis using ASL with multiple post-labeling delays (26). In contrast, acquisition of ASL in the present case was performed with a single post labeling delay; nonetheless, it allowed detection of severe intralesional hypoperfusion core as well as peripheral hyperperfusion. Peripheral contrast enhancement surrounding the infarct area has been described in dogs as indicative for vascular leakage (27, 28). However, the evidence of supernumerary acquired arterial branches visible on the left middle cerebral artery together with PWI findings suggest peripheral hyperperfusion of the lesion instead. This may be an indicator for early reperfusion (29, 30). Susceptibility imaging enables detection of venous blood, hemorrhage or iron storages (31). Identification of the thrombus has been reported to be possible in small vessels by means of SWI due to the paramagnetic properties of the iron contained within it (32). In contrast, a focal signal void in the area of the left striate artery was not visible in the present case.

Collateral blood supply has been shown in an experimental canine study using corrosion casting 8 weeks after BCAL (1). However, the present report evidences the utility of MRA for evaluating collateral blood supply after BCAL *in vivo*. Blood flow in both common carotid arteries was absent in the caudal cervical region and showed increased signal intensity again at the level of C2, branching normally cranial to this level. An extensive vascular network was seen in the vicinity of the ligation sites of the common carotid arteries, not only paralleling these arteries but also extending between the vertebral arterial system and the carotids and extending rostrally. Superficial arterio- arterial collaterals between the caudal auricular and the cranial thyroid arteries and the deep collateral vessel system connecting both vertebral arteries and the common carotid arteries were identified, consistent with the abovementioned experimental study (1). Numerous arterial- arterial anastomoses were depicted in both MRA techniques, however we could not identify neither a separate vascular network resembling the anastomosis between maxillary and the internal carotid arteries nor could we identify an anastomotic connection between the internal carotid and the ascending pharyngeal artery as described in the corrosion casting model (1). Clear visualization of the striate arteries was also not possible using a 3-Tesla scanner. This is consistent with previous anatomical studies applying the same MRA techniques using a 1.5-Tesla scanner (13, 14).

Albeit performing MRI according to the most recent recommendations and including new functional techniques, MRI findings fail to explain the pathomechanism behind the occurrence of the ischemic infarct and proving that the ischemic infarct reported in this case is a direct consequence of BCAL is not possible. However, the patient was a young and healthy working dog and besides risk factors related to the traumatic acute bleeding, no chronic diseases predisposing to stroke were identified. Determining a cause for ischemic infarcts is not possible in up to 50% of canine ischemic stroke patients (27, 33, 34). Similarities to human patients with postoperative stroke after carotid artery ligation highlight a possible causality (35).

Platelet inhibition and anticoagulant therapies where only started after neurological deterioration and for this reason we do not know if this infarct could have been prevented by initiating this treatment directly after surgery. No recommendation exists in veterinary medicine regarding medical thrombus prevention in this type of surgery and it is beyond of the scope of a single case report to give treatment recommendation. Still this case report should raise awareness for strokes as possible complication after vascular ligation surgery in dogs. The use of anticoagulant therapy in the postoperative period should however be considered on a case by case basis weighing possible risks and benefits.

## Conclusion

Collateral blood supply to the brain can be visualized in MRA as soon as 5 days after BCAL. Ischemic infarct due to thromboembolism must be considered as a possible complication of this procedure.

## Data Availability Statement

All datasets generated for this study are included in the article/Supplementary Material.

## Ethics Statement

Ethical review and approval was not required for the animal study because data were collected retrospectively. Written informed consent was obtained from the owners for the participation of their animals in this study.

## Author Contributions

LK, AW-L, and KB contributed to conception and design of the study and wrote the first draft of the manuscript. LK collected clinical data. IL and AW-L wrote the imaging part of the manuscript. IL and AV wrote sections of the manuscript. AP, AV, AW-L, and KB corrected and finalized the manuscript. All authors contributed to manuscript revision, read, and approved the submitted version.

## Conflict of Interest

The authors declare that the research was conducted in the absence of any commercial or financial relationships that could be construed as a potential conflict of interest.

## Supplementary Material

Supplementary Figure 1 Pre **(A)** and post contrast **(B)** Transverse CT images in low frequency reconstruction at the level of the orbital fissures. Severe amount of heterogeneous soft tissue attenuating material within the nasopharynx, with few gas inclusions. Crescent-shaped contrast pooling medial from the right pterygoid process after iodine-based contrast medium injection (white arrow).
