# Supplementary figures and images for: Striate Artery Infarct After Bilateral Carotid Artery Ligation (BCAL) in a Dog: A Multimodal MRI Study
Source: Front Vet Sci. 2020 Sep 18;7:580256. doi: 10.3389/fvets.2020.580256 (PMC7533532; doi:10.3389/fvets.2020.580256)

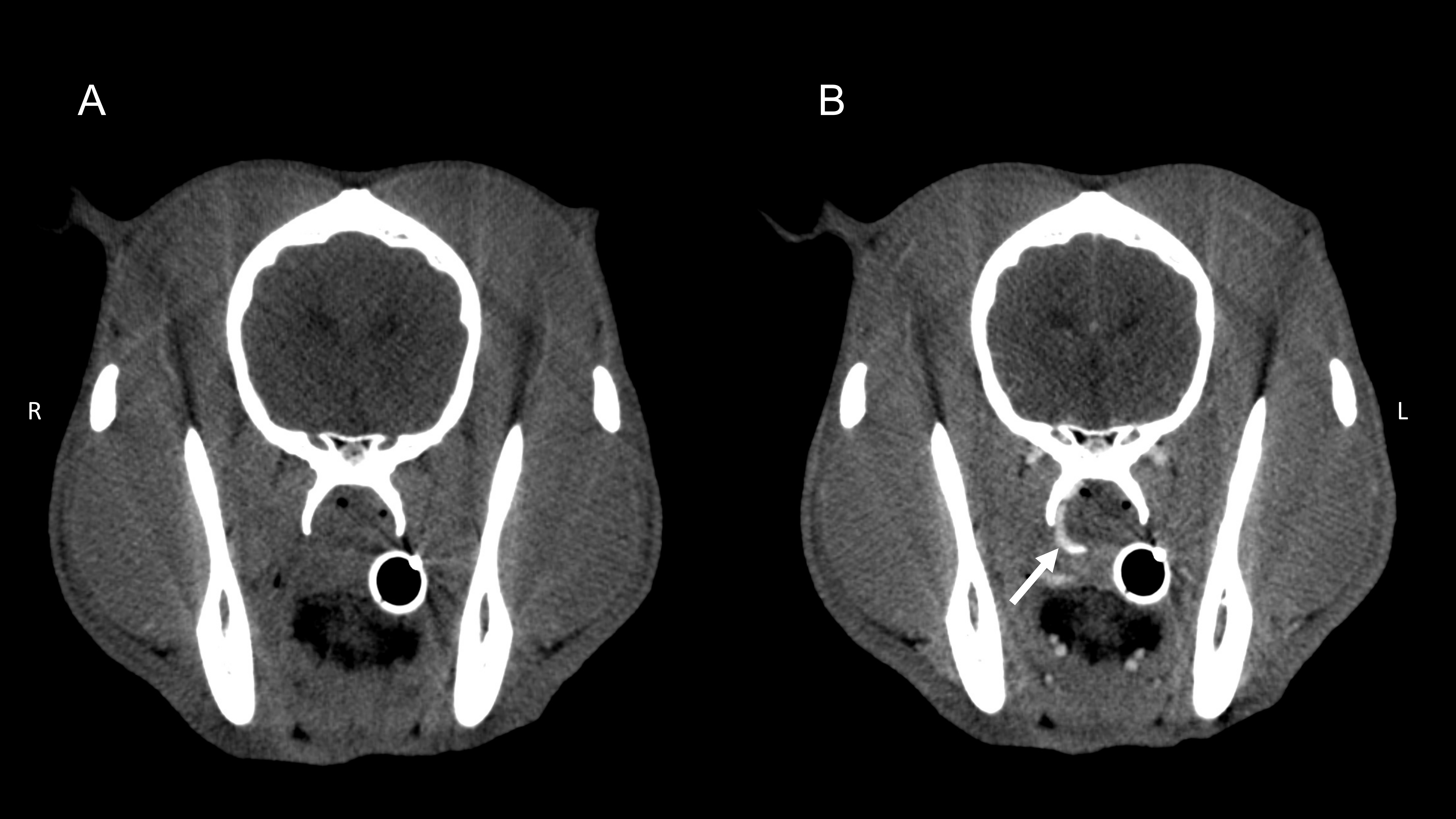

Supplement: Supplementary Figure 1 — Pre (A) and post contrast (B) Transverse CT images in low frequency reconstruction at the level of the orbital fissures. Severe amount of heterogeneous soft tissue attenuating material within the nasopharynx, with few gas inclusions. Crescent-shaped contrast pooling medial from the right pterygoid process after iodine-based contrast medium injection (white arrow). [file Image_1.TIFF]
